# *Staphylococcus aureus* entanglement in self-assembling β-peptide nanofibres decorated with vancomycin†

**DOI:** 10.1039/d0na01018a

**Published:** 2021-03-24

**Authors:** Jennifer A. E. Payne, Ketav Kulkarni, Thierry Izore, Alex J. Fulcher, Anton Y. Peleg, Marie-Isabel Aguilar, Max J. Cryle, Mark P. Del Borgo

**Affiliations:** Infection and Immunity Program, The Monash Biomedicine Discovery Institute, Department of Biochemistry and Molecular Biology, Monash University Clayton Victoria 3800 Australia jennifer.payne@monash.edu max.cryle@monash.edu; EMBL Australia, Monash University Clayton Victoria 3800 Australia; The ARC Centre of Excellence for Innovations in Peptide and Protein Science, Monash University Clayton Victoria 3800 Australia; Department of Biochemistry and Molecular Biology, Monash University Clayton Victoria 3800 Australia mibel.aguilar@monash.edu; Monash Micro Imaging, Monash University Clayton Victoria 3800 Australia; Infection and Immunity Program, Monash Biomedicine Discovery Institute, Department of Microbiology, Monash University Clayton Victoria 3800 Australia; Department of Infectious Diseases, The Alfred Hospital, Central Clinical School, Monash University Melbourne Victoria 3004 Australia; Department of Pharmacology, Monash University Clayton Victoria 3800 Australia mark.delborgo@monash.edu

## Abstract

The increasing resistance of pathogenic microbes to antimicrobials and the shortage of antibiotic drug discovery programs threaten the clinical use of antibiotics. This threat calls for the development of new methods for control of drug-resistant microbial pathogens. We have designed, synthesised and characterised an antimicrobial material formed *via* the self-assembly of a population of two distinct β-peptide monomers, a lipidated tri-β-peptide (β^3^-peptide) and a novel β^3^-peptide conjugated to a glycopeptide antibiotic, vancomycin. The combination of these two building blocks resulted in fibrous assemblies with distinctive structures determined by atomic force microscopy and electron microscopy. These fibres inhibited the growth of methicillin resistant *Staphylococcus aureus* (MRSA) and associated directly with the bacteria, acting as a peptide nanonet with fibre nucleation sites on the bacteria observed by electron microscopy and confocal microscopy. Our results provide insights into the design of peptide based supramolecular assemblies with antibacterial activity and establish an innovative strategy to develop self-assembled antimicrobial materials for future biomedical application.

## Abstract 2

Peptides containing vancomycin were able to self-assemble into antimicrobial 2D scaffolds.

## Graphical abstract

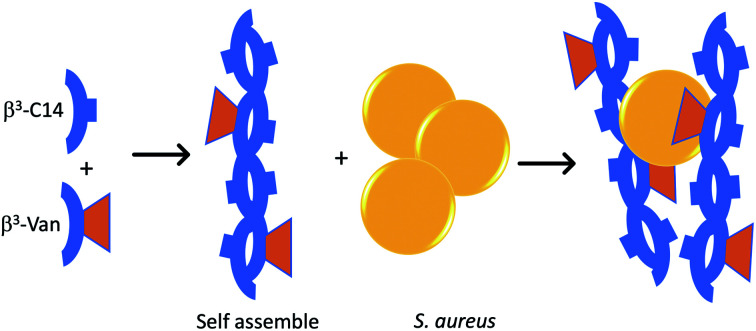

## Introduction

The rapid emergence of antimicrobial resistance represents a global health crisis where there are dwindling therapeutic options.^1–3^ Additionally, biofilms establish on medical surfaces, in which microbial communities are embedded in an extracellular matrix.^4^ This prevents the effective penetration and use of antibiotics, meaning biofilms are over 1000-fold resistant to current antibiotics.^5^ Thus, the development of antimicrobial therapies that not only kill the microbe,^6^ but also offer innovative strategies to control and prevent infection and biodevice fouling are urgently required.^7–9^ In response to these challenges, there have been a number of studies that have investigated the potential of biomaterials as effective antimicrobial agents and coatings.^2,3^ These materials are generally designed to mimic the structural properties of cationic antimicrobial peptides, which lyse membranes *via* binding and penetrating anionic microbial membranes. The resulting antimicrobial materials are synthesised either through polymerisation or are derived from the supramolecular self-assembly of naturally occurring or synthetic antimicrobial molecules.^2,10–12^

Beyond these, one novel approach of particular interest from the materials science perspective is the design of new materials that can entrap and kill bacterial pathogens by formation of antimicrobial fibres into nanonets. The human innate immune response provides inspiration for this approach, as it produces nanonets that entangle invading microbes in a fibrous mesh of peptides and DNA, preventing bacterial dissemination and bringing microbes into contact with high local concentrations of antimicrobials.^13^ These DNA nanonets are termed Extracellular Traps (ETs) and are composed of nuclear and mitochondria DNA arranged in ∼17 nm diameter filaments decorated with globular domains of ∼50 nm.^14,15^ The antimicrobial activity of these fibres arises from embedded granular and specific cytoplasmic proteins, which confer multiple functions including recognition, direct killing, degradation of virulence factors, and remodelling of the surrounding tissue.^13^ These ETs are produced within minutes by innate immune cells such as macrophages (METs)^16^ and neutrophils (NETs).^14^ While these ETs play a critical role in preventing infection, microbes have evolved strategies to escape the deadly entanglement of ETs by producing nucleases to degrade the DNA ETs, an essential process for their virulence.^17–19^ Another example of nanonets in immunity are the peptide-based nanofibres resulting from the self-assembly of antimicrobial peptide monomers.^20^ For example, the monomeric form of human alpha defensin 6, HD6 (3.7 kDa), exhibits poor antimicrobial activity but kills bacteria when self-assembled into fibril nanonets.^21,22^

Given that both DNA and peptide nanofibre strategies result in the entrapment of the bacteria and direct antimicrobial activity, we sought to develop an artificial peptide-based nanonet using self-assembling β-peptides. A key feature of β-peptides, which adopt a 14-helical conformation, is that the backbone is responsible for self-assembly, allowing side chains to be functionalised without compromising assembly.^20,23^ Compared to other peptide-based self-assembling materials, *N*-acetylated β^3^-peptides form nanofibres in solution or on surfaces and do not require a specific peptide sequence to self-assemble.^20,24,25^ Bespoke amino acids can thus be developed and integrated within the β^3^-peptide sequence without compromising self-assembly and makes them ideal for biomaterial development.

**Fig. 1:**
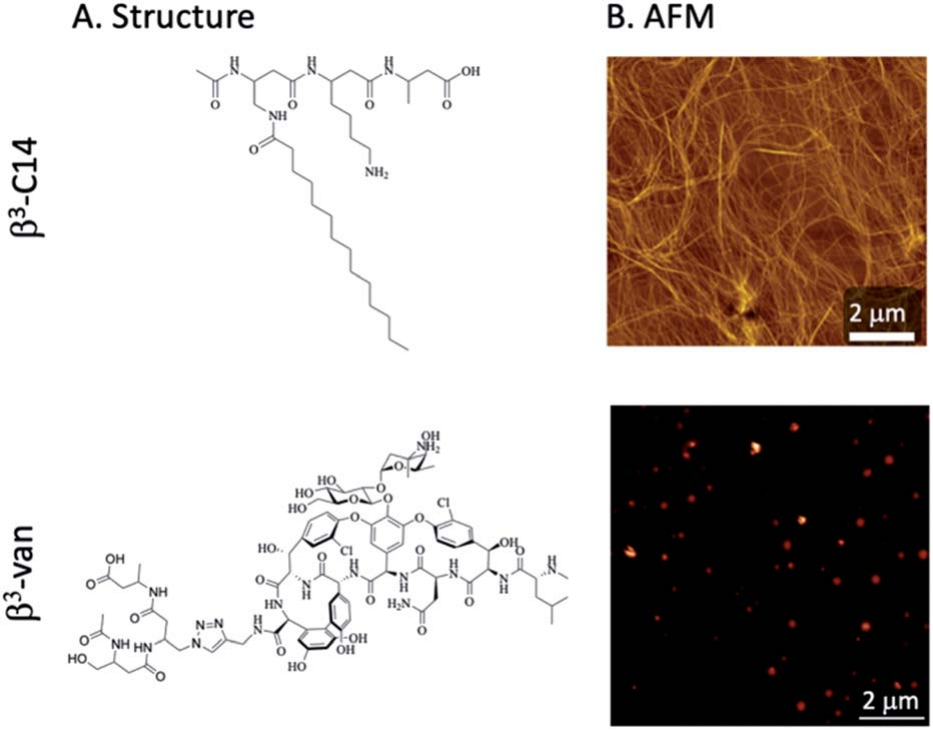
Structure of two β^3^-peptides. (A) The self-assembling C14 lipidated β^3^-peptide (β^3^-C14, *AcN*-βX(C14)–βLys–βAla, 5) and a β^3^-peptide linked to the glycopeptide antibiotic vancomycin (β^3^-Van 3, *AcN*-β-Ser–βX(Vanc)–βAla). (B) AFM imaging of fibres following the self-assembly of β^3^-C14 (5) at 0.4 mM, fibres were not formed by β^3^-Van (3).

In this study, we have created a self-assembling β^3^-peptide-based nanofibrous material that is decorated with the glycopeptide antibiotic vancomycin. Vancomycin was chosen as it binds directly to the cell wall of Gram-positive bacteria, thus allowing potential activity when displayed on the surface of the self-assembled nanomaterials, as opposed to an antimicrobial agent that acts against a cytosolic bacterial target. We successfully synthesised a β-tripeptide-vancomycin conjugate (β^3^-Van, 3) and showed that its co-assembly with a lipidated β-tripeptide (β^3^-C14, 5) results in fibrous assemblies that allow for precise control over exposure of the glycopeptide antibiotic (Fig. 1). Control of the level of vancomycin loading within these biomaterials resulted in defined structural features within the 2D co-assemblies, observed using high resolution microscopy (AFM) imaging. Furthermore, these fibres displayed vancomycin on their surface, as detected by an AFM probe coated in vancomycin binding motif, and display direct antimicrobial activity against Gram-positive bacteria.

## Results and discussion

### β-Peptide design and synthesis

The aim of this study was to synthesise an antimicrobial peptide-based biomaterial that mimicked the bacteria-encapsulating properties of naturally occurring nanonets. Thus, we designed a novel fibrous material comprised of two different tri-β-peptides (β^3^-peptides). β-Peptides that are comprised solely of β-amino acids adopt well-defined helical structures stabilised by hydrogen bonding,^26,27^ the most commonly observed being either a 14- or 12-helical conformation. Of relevance to the present study, the 14-helix contains 14 atoms in the hydrogen-bonded ring, and is stabilised by hydrogen bonding between the main chain amide proton (HN) at position i and the carbonyl (CO) at position i + 2.^26^ This 14-helix corresponds to ∼3 residues per turn, which results in the alignment of the side chains of every fourth residue directly along one face of the helix.^26^ We have previously demonstrated that this molecular symmetry induces the supramolecular self-assembly of *N*-acetylated β^3^-peptides containing at least three β^3^-amino acids *via* a head-to-tail H-bonded motif.^20,23^

More recently, we demonstrated the controlled self-assembly of lipidated *N*-acetyl β^3^-peptides to give a range of well-defined fibres and nano-ribbons.^28^ Also, these materials can form biocompatible hydrogels for cell culture^29–32^ and *in vivo* applications.^33,34^

**Scheme 1:**
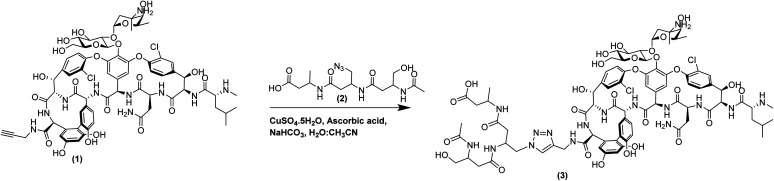
Conjugation between propargyl amide vancomycin (1) and β^3^-tripeptide (2) *via* click chemistry to synthesise β^3^-Van (3).

The activity of these β^3^-peptide materials has been modulated through the introduction of novel β-amino acids containing cell adhesion epitopes and fluorophores for localisation studies. We therefore aimed to exploit the facile fibre formation of β-peptides to prepare an antimicrobial β-peptide fibre. Our strategy involved the use of an *N*-acetylated β^3^-peptide containing a tetradecyl lipid chain at residue 1 (β^3^-C14, 5, Fig. 1A) as the self-assembling template. While it is possible to then conjugate a vancomycin molecule to residue 3, the resulting peptide is very hydrophobic and extremely difficult to handle. We therefore replaced the C14 chain with a vancomycin molecule to give the new β-peptide 3 (β^3^-Van, Fig. 1A) using click chemistry (71% yield), which is detailed in the Scheme 1.

Click chemistry is a well-established ligation method that utilises the chemo-selectivity of copper catalysed azide–alkyne cycloaddition reactions (CuAAC) and results in rapid, irreversible and regioselective formation of 1,4-disubstituted 1,2,3-triazoles under mild conditions. Vancomycin conjugates have been previously developed using this chemistry by ligating small molecules^5^ to antimicrobial peptides to develop heterodimer antibiotics,^35^ or homodimerisation of vancomycin using tethers.^36^ Derivatisation of glycopeptide antibiotics, such as vancomycin, is also possible *via* incorporating residues with an alkyne side chains into the peptide backbone.^37^ However, for this study we used vancomycin, which can be potentially derivatised at three functionally distinct sites: a primary amine on the vancosamine amino sugar; C-terminal carboxylic acid; or the secondary amine of the N-terminal *N*-methylated leucine residue. The N-terminal residue of vancomycin is crucial to its potency, as it binds to the d-Ala–d-Ala terminus of the peptidoglycan of Gram-positive pathogens inhibiting cell wall biosynthesis and this has been confirmed by the lack of antimicrobial activity of desleucyl-vancomycin.^38^ The sugar groups play an important role in the dimerisation of vancomycin, which is believed to promote its antimicrobial actions by formation of a ‘chelate’ with the peptidoglycan ligands.^39^ Therefore, it is prudent to derivatise vancomycin *via* the C-terminal carboxylic acid, as it is the least disruptive to its key interactions with the bacterial cell wall, thereby not compromising its mode of action and minimising loss of antimicrobial activity. Indeed, this is a common feature of second-generation antibiotics derived from vancomycin. Thus, a C-terminal propargyl amide modified vancomycin molecule was synthesised, which could then be conjugated through the azide containing β^3^-peptide *via* a copper-catalysed Huisgen 1,3-cycloaddition reaction.

The azide–alkyne cycloaddition is copper catalysed and requires the catalyst, prepared with an appropriate chelating ligand, to be maintained in the Cu(i) oxidation state, which is commonly achieved by the use of an *in situ* reducing agent such as sodium ascorbate. To synthesise the vancomycin click-conjugates, precedence in the literature is given to the classical click-reaction conditions using CuSO_4_, ascorbate and aqueous solvents with either ^*t*^BuOH, DMSO, DMF or MeOH in water. However, the major drawback of the click-reaction is the copper-mediated formation of reactive oxygen species (ROS), or the reactive by-products within the reaction mixture, such as dehydroascorbate, which is further hydrolysed to form 2,3-diketogulonate, leading to the oxidative degradation especially of proteins or peptides.^40,41^ To overcome these limitations, the previously reported reaction conditions were optimised to either decrease the reaction time, perform the reaction under anaerobic conditions, or generate a stable Cu(i) catalyst. The addition of a ligand, such as those belonging to the tris(triazolylmethyl)amine family, within the reaction mixture stabilises the Cu(i) species.^42^ Additionally, the use of acetonitrile as a co-solvent acts to accelerate the reaction by stabilising the Cu(i) intermediate and removes the requirement for the use of additional ligands.^43^ The reaction conditions were thus optimised using acetonitrile as a surrogate ligand under aqueous conditions to stabilise the Cu(i) species, tailored to the polarity and solubility of the azide and alkyne substrates, while maintaining CuSO_4_ as the copper source and sodium ascorbate as the reducing agent. These pseudo-ligandless conditions were also compatible with the peptide purification method as the reaction mixture could be purified immediately, using reversed phase HPLC, to afford the vancomycin conjugated β-tripeptide (β^3^-Van, 3) in high yield.

### β-Peptide fibre formation

**Fig. 2:**
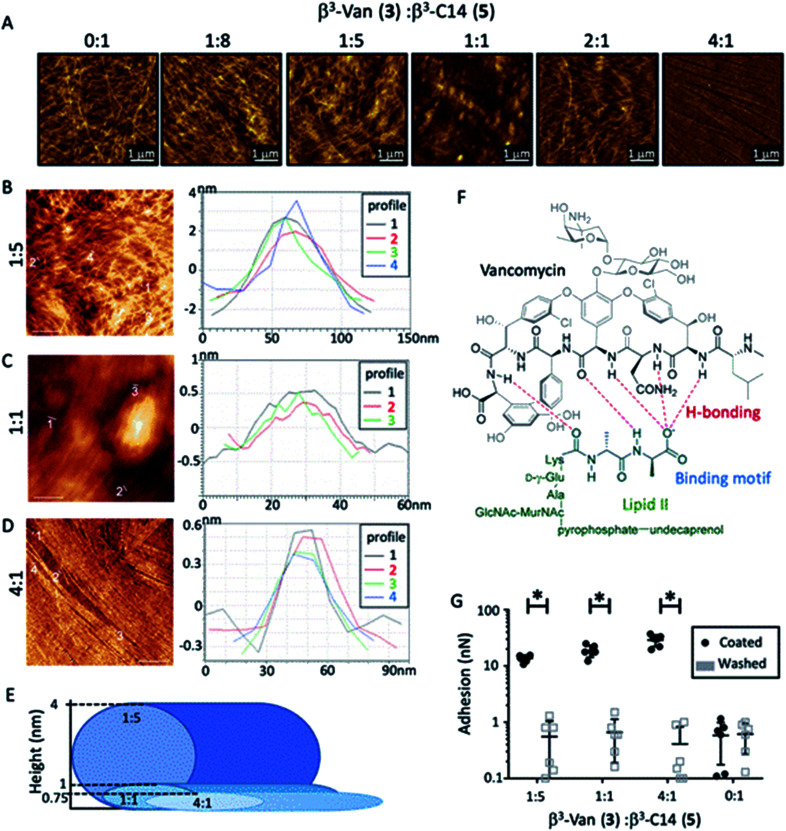
Mixing the two β^3^-peptides (0.4 mM) resulted in nanofibres that incorporated vancomycin. (A) AFM revealed the fine structures of the nanofibres composed of different ratios of β^3^-Van (3) : β^3^-C14 (5). AFM mapping of the fibres quantified the increasing fibre height observed with increasing amounts of β^3^-C14 (5) (B–D) with the average diameter of these nanorods depicted in (E). Scale bars 1 μm. (F) Vancomycin binds to lipid II (green) in the *S. aureus* cell wall. In particular, the d-Ala–d-Ala motif (highlighted in blue) is bound through hydrogen bonds (red) to the vancomycin β^3^-peptide backbone (black). (G) Vancomycin was detected within the nanofibres by coating the AFM probe with this d-Ala–d-Ala binding motif (6). The force required to remove the probe from the fibres containing β^3^-Van (3) was 15 times greater than lipidated fibres (0 : 1) alone (black bars). Washing the probe of d-Ala–d-Ala (6) removed this binding (light grey bars). Error bars are SD, *n* = 6, with 2-way ANOVA with a post-hoc Tukey's test *P* < 0.001.

The formation of fibres was determined by atomic force microscopy (AFM) analysis of a series of mixtures of β^3^-C14 (5) and β^3^-Van (3) (Fig. 2A and B). β^3^-C14 (3) (*i.e.* β^3^-Van : β^3^-C14 = 0 : 1) showed the formation of a nanofibrous network consistent with previous studies,^28,32^ with individual fibres approximately 10 nm in diameter. However, the supramolecular self-assembly of these peptides was perturbed by the inclusion of vancomycin onto a sidechain, as no fibre structure was observed for 100% β^3^-Van by AFM (Fig. 1B). Instead, we observed the formation of regular sized aggregates of 30–40 nm. This change in the self-assembly properties of the peptides may be influenced not only by the large size of the vancomycin payload, but also the dimerisation of vancomycin that occurs through the sugars moieties,^44,45^ which may impede the head-to-tail self-assembly of the β-peptide.

The effects of increasing amounts of β^3^-Van (3) were then investigated using the β^3^-Van : β^3^-C14 ratios of 1 : 8, 1 : 5, 1 : 1, 2 : 1, 4 : 1 and 1 : 0. While β^3^-Van (3) alone did not produce fibres, co-assembly of the two β^3^-peptides formed nanofibres with distinct morphologies (Fig. 2A). Increasing the β^3^-Van (3) present in the fibre reduced the bundling of these fibres, as there was a decrease in the average fibre height and a greater alignment of the fibres (Fig. 2A–D). AFM revealed the presence of slender, straight fibres with the 4 : 1 ratio of β^3^-Van (3) to β^3^-C14 (5). The height of these fibres was ∼0.75 nm, suggesting that these fine fibres are composed of a single nanorod (Fig. 2D). Furthermore, the nanorods appear to be tightly packed and have a flat surface. The supermolecular structures that vancomycin forms beyond the dimer, into tetrameric and hexameric forms,^46^ may be influencing the fibres to align and forming the striking alignment observed in the 4 : 1 ratio. A schematic representation of the fibrous dimensions observed can be seen in Fig. 2E.

AFM force mapping was used to confirm the presence and accessibility of the vancomycin moiety on the surface of these co-assembled nanofibres by coating the AFM probe with d-Ala–d-Ala (6). This dipeptide motif is the binding target of vancomycin within the lipid II cell wall precursor of the bacterial cell wall (Fig. 2F), with complex formation between vancomycin and the d-Ala–d-Ala dipeptide leading to the antibiotic activity observed for vancomycin.^45,47^ The force required to remove the dipeptide-coated (d-Ala–d-Ala (6)) AFM probe from the fibres containing β^3^-Van (3) was 15 times greater than for the β^3^-C14 (5) fibres alone (Fig. 2G), while testing adhesion of the probe after washing removed this interaction. These experiments demonstrated that the vancomycin payload within the β^3^-peptide fibres is exposed on the surface of the fibres, and we then determined if vancomycin was functional as an antibiotic agent following the self-assembly process.

### Antibacterial activity of β-peptide fibres

**Fig. 3:**
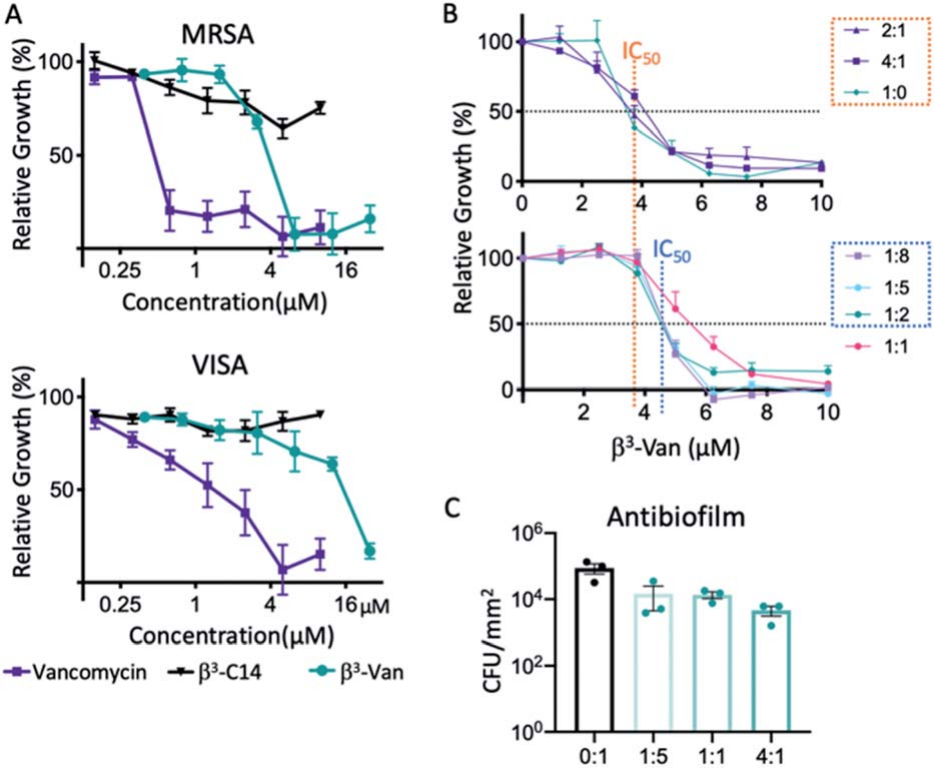
Fibres with embedded vancomycin have antimicrobial activity against *S. aureus*. (A) The growth inhibition activity of the peptides was tested against two antibiotic resistant clinical isolates of *S. aureus*, methicillin resistant (MRSA), and vancomycin intermediate sensitivity (VISA). Using the microbroth dilution assay β^3^-C14 (5) had no activity at the concentrations tested, while β^3^-Van (3) inhibited growth of both strains at higher concentrations than vancomycin alone. Error bars are SEM, *n* = 3 biological replicates. (B) The growth inhibition activity of the mixed nanofibres against MRSA was determined using a microbroth dilution assay. The orange dotted line marks the average IC_50_ of the mixtures containing higher amounts of β^3^-Van (3) (2 : 1, 4 : 1 – β^3^-Van : β^3^-C14) compared to the blue dotted line marking the increased IC_50_ value for the mixtures containing less β^3^-Van. Error bars are SEM, *n* = 3 biological replicates. (C) Nanofibres assembled on a glass surface at 0.4 mM, reduced *S. aureus* biofilm formation with increasing ratios of β^3^-Van (3) : β^3^-C14 (5). Error bars are SD, results are representative of 3 biological replicates.

The antimicrobial activity of selected β-peptide fibres was then assessed in a microbroth dilution assay against both clinical antibiotic resistant *S. aureus* isolates – a methicillin resistant *S. aureus* (MRSA) and a vancomycin-intermediate resistance *S. aureus* (VISA) strain.^27^ The fibres comprising only β^3^-C14 (5) exhibited no antimicrobial activity (Fig. 3A). However, β^3^-Van (3), which was derived from the attachment of the β^3^-peptide onto the carboxyl terminus of vancomycin, exhibited significant activity against both isolates, albeit lower than for free vancomycin (Fig. 3). Specifically, β^3^-Van (3) exhibited a 6-fold reduction in antimicrobial activity against the MRSA isolate, and 4-fold against the VISA isolate compared to vancomycin. A loss in antimicrobial activity relative to unmodified vancomycin is likely due to the presence of the β^3^-peptide attachment on the C-terminal region of vancomycin. However, as discussed previously, the choice of linkage through the carboxyl terminus of vancomycin rather than the primary amine on the sugar or the *N*-methylated amine was adopted to minimise further loss in activity if attached through alternate sites.^48^

We then analysed the antimicrobial activity of different mixtures of peptides β^3^-Van (3) and β^3^-C14 (5). The minimal inhibitory concentration (MIC) was 6.25 μM for all peptide ratios, except for the 1 : 1 (3/5) mixture where there was an increase in MIC to 7.5 μM. There were differences in the concentration that inhibited 50% growth (IC_50_), with mixtures containing the higher amounts of β^3^-Van (3) of 2 : 1 and 4 : 1 (3/5) had similar activity to the β^3^-Van (3) alone (1 : 0; 3/5, Fig. 3B, upper). However, ratios of 1 : 8, 1 : 4, and 1 : 2 resulted in an increase in IC_50_ compared to the β^3^-Van (3) alone (Fig. 3B, lower), suggesting a reduction in antimicrobial activity at lower concentrations of these ratios despite having similar MIC values.

The antibiofilm activity of selected mixtures was then examined at higher concentrations of 0.4 mM, which is known to form fibres. The co-assembled fibres were coated onto a glass surface, and the presence of fibres with the same morphology was confirmed by AFM (data not shown). A biofilm forming *S. aureus* strain was then grown for 24 h on the surface of these nanofiber coated glass surfaces. Biofilm growth was reduced relative to the presence of β^3^-Van (3) in the fibres, with the 4 : 1 resulting in a 19-fold reduction in *S. aureus* CFU compared to the β^3^-C14 fibres alone (Fig. 3C).

**Fig. 4:**
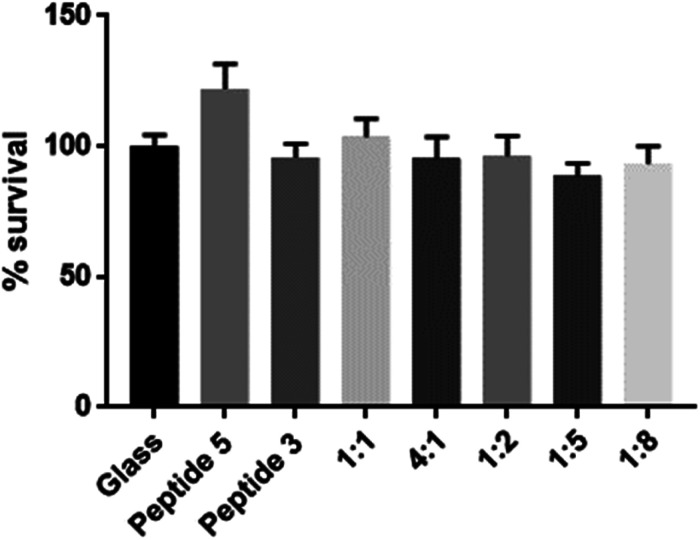
Coverslips coated with various scaffolds were seeded with HaCaT cells, incubated for 48 hours and assessed for viability *via* an MTS assay. No discernable cytotoxicity was detected. Experiments were performed in triplicate, *n* = 3, error bars are SD.

To assess biocompatibility, we again coated coverslips as described previously and seeded the glass coverslips with human keratinocytes (HaCaT cells, kindly provided by A/Prof J. Frith, Dept Materials Science & Engineering, Monash University). An MTS assay was used to assess cell viability following 48 h of incubation. As shown in Fig. 4, no cytotoxicity was apparent with any of the scaffolds tested.

**Fig. 5:**
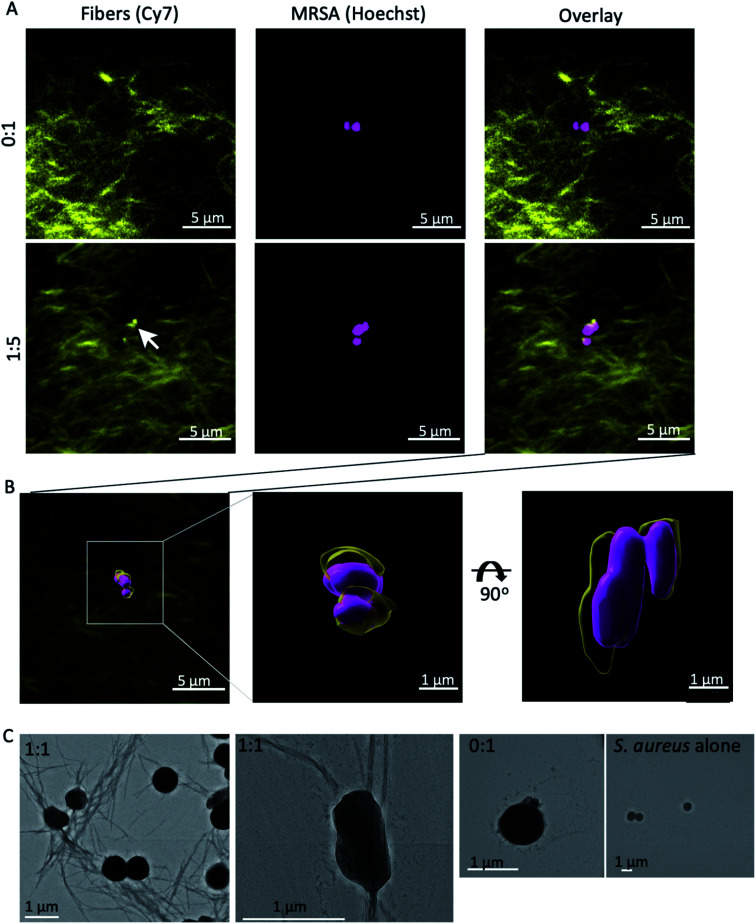
Nanofibres were imaged in the presence of MRSA by confocal and electron microscopy. (A) Nanofibre visualisation was achieved by including 0.1% fluorescently labelled β^3^-C14 (4) in both ratios, and MRSA by Hoechst staining using a Leica SP8 with HyD detector. A group of nanofibres (arrow) localised with MRSA when β^3^-Van (3) was present and was not observed with the β^3^-C14 (5) fibres alone. (B) The surfaces of both fibres and MRSA were modelled using 3D rendering software Imaris with surface rendering applied locally over the site of nucleation. (C) Fibres were incubated with MRSA and then washed, before being imaged by electron microscopy. Fibres were only observed in washed samples when vancomycin was present in the fibres.

We next examined the interaction the self-assembling mixed fibres with MRSA by confocal microscopy. The nanofibres were visualised by including 0.1% fluorescently labelled β^3^-C14 (4) in each peptide mixture, with Hoechst staining of the nuclei of MRSA (Fig. 5A and B). This fluorescent peptide has been used previously to visualise fibre formation^34^ and a synthetic protocol can be found in ESI.†

The presence of β^3^-Van (3) resulted in the apparent association of fibres with MRSA (ESI Video 1†), which was absent when fibres lacked vancomycin (ESI Video 2†). This association between the β^3^-Van containing fibres and MRSA was further confirmed by electron microscopy (Fig. 5C). Nanofibres at 100 μM were incubated with MRSA, washed and then imaged by EM. Fibres were observed by EM in association with MRSA when the fibres contained β^3^-Van (3) (Fig. 5C) and were absent with fibres comprised of only β^3^-C14.

These results suggest that MRSA may either act as a nucleation site for the self-assembly of the β^3^-Van containing fibres or strongly associate with the exposed vancomycin moieties during fibre formation. This would be similar to HD6 that nucleates on bacterial cell surface proteins to form nanonets *in vivo*.^14,22^ Our mixed β^3^-peptide fibres therefore entrap and kill MRSA in a manner reminiscent of the DNA ETs and HD6 nanonets utilised by the innate immune system.^14,22^

Incorporation of antimicrobial activity into the nanostructure of a simple tripeptide by self-assembly may have important applications for novel antimicrobial coatings, as it offers a novel approach to tailor the material for different antimicrobial applications by modifying the peptide backbone to incorporate different functionalities. However, this is the first example of a functionalisation that has inhibited the self-assembly of β^3^-peptides, which was overcome by the formation of a composite material. This strategy could be further exploited by incorporating modular functionalities into the β^3^-peptide fibre, which would allow extracellular traps to be decorated with enzymes displaying antimicrobial activities. Since lipidated β-peptides are also able to form hydrogels,^28,30,32^ the potential of these peptides to yield antimicrobial hydrogels would have wide application in areas such as wound management. Moreover, β^3^-peptide materials could provide a scaffold within the wound allowing migration of cells into the gel^34^ while also inhibiting bacterial growth, which are two important properties for the treatment of chronic wounds. The incorporation of the bulky vancomycin into the fibres also demonstrates the possibility of incorporation of other large functionalities, such as growth factors to promote wound healing, into such fibre assemblies in future.

## Conclusions

We have developed the first synthesis of a β^3^-peptide conjugated with vancomycin, which undergoes supramolecular self-assembly to yield a fibrous material with vancomycin on the fibre surface and yielding antimicrobial activity. The presence of vancomycin on the periphery of the nanofibre increases the effective local concentration compared to soluble antibiotic, and is likely the driving force for improved antibiofilm activity. Our co-assembled nanofibres represent a strategy to design smart materials, and underpins further development, such as the introduction of a broader range of antibiotics for different microbial systems and other functionalities for wound-healing applications. This study also demonstrates the versatility of these biocompatible β-peptide materials where the long-term chemical and proteolytic stability is combined with the ability to biofunctionalise to provide a powerful template for smart material design.

## Experimental

### Chemicals

Commercially available chemicals were purchased from Sigma-Aldrich Co. (St. Louis, Missouri, USA), Merck KGaA (Darmstadt, Hesse, Germany), GL Biochem (Shanghai, China). (9-Fluorenylmethyloxycarbonyl)amino-4-azido-butanoic acid was purchased from Iris Biotech Gmbh (Marktredwitz, Germany). ^i^Pr_2_EtN was distilled from CaH; DMF was stored over 4 Å molecular sieves.

### Peptide synthesis

All peptide synthesis and purification are described in the ESI.†

### NMR

^1^H NMR spectra were obtained at 400 MHz on Bruker Avance III 400 spectrometer (Bruker BioSpin Corporation, Billerica, Massachusetts, USA and Varian Inc., Palo Alto, California, USA). The residual solvent peak (3.31 ppm for CD3OD) was used as an internal reference.

### Mass spectrophotometry

Mass spectra were acquired using an Agilent 1100 MSD SL ion trap mass spectrometer (Agilent Technologies, Santa Clara, California, USA). High resolution mass spectra were recorded using an Agilent G1969A LC-TOF system (Agilent Technologies, Santa Clara, California, USA), with reference mass correction and a capillary voltage of 4000 V for ESI; and an Agilent 7200 Q-TOF GCMS system.

### High performance liquid chromatography

Preparative High-Performance Liquid Chromatography (HPLC) was performed using an Agilent 1200 series HPLC system. Samples were injected onto a reverse-phase preparative (C18, 300 Å, 5 μm, 10 mm × 250 mm) column and detected at wavelengths of 214 nm and 280 nm. Analytical HPLC was performed using an Agilent 1100 series HPLC system. The samples were injected onto a reverse-phase analytical (C18, 300 Å, 5 μm, 4.6 mm × 150 mm) column and detected at a wavelength of 214 nm.

### Preparation of peptide mixtures

Peptides 3 and 5 were weighed out, dissolved in water and combined to give the following molar ratios; 1 : 0, 0 : 1, 1 : 8, 1 : 5, 1 : 1, 2 : 1, 4 : 1. These solutions were then lyophilised and dissolved immediately before use.

### Atomic force microscopy imaging of nanofibres

Prepared peptide mixtures of 3 and 5 were re-dissolved in water at 0.4 mM and 2 μL was placed on a freshly peeled mica surface glued to a 12 mm metal AFM specimen disc. The mica was covered with a Petri dish to avoid any possible contamination and air dried in a fume hood. Prior to microscopy, each sample was further dried with a gentle stream of N_2_ gas. Morphological characterization was carried out using a Bruker Fastscan AFM (Bruker, Santa Barbara, CA, USA). Images were obtained using peakforce-tapping mode with Fastscan C probes (Bruker, Santa Barbara, CA, USA) with a nominal spring force constant of 0.8 N m^−1^. Topographic and phase were simultaneously obtained at a resolution of 512 × 512 using a scan frequency of 2 Hz with typical scan sizes 5 μm × 5 μm. 15 images per sample were taken and images were processed using Gwyddion 2.45 software. Fibre dimensions were quantified by using line sectioning and height dimensions were averaged as previously described.^28^

### AFM measurements of adhesion

To assess the presentation of vancomycin on the surface and the ability to recognise the d-Ala–d-Ala motif, Fastscan C probes were coated with peptide 6 by submersing the AFM probe in a concentrated solution of 6 (1 mg mL^−1^). The probe was allowed to dry by evaporation using a gentle stream of N_2_. This process was repeated to ensure a complete coating of the tip surface. The samples described above were then re-imaged using the coated probe using quantitative nanomechanical mapping (Peakforce-QNM). Topographic, phase and adhesion were simultaneously obtained at a resolution of 512 × 512. The cantilever spring constant was calibrated before each experiment. Finally, the probe was submerged in boiling water and dried three times and each sample then re-imaged using the above conditions. 6 images per sample were taken and data processing was performed using the commercial Nanoscope Analysis software (Bruker AXS Corporation). Adhesion was determined by the average raw value (in nN) along 8 lines drawn onto each of the 6 images in an identical pattern, and the average plotted.

### Bacteria strains

The American Type Culture Collection strain (ATCC 29213) and paired clinical isolates of *S. aureus* – MRSA (genotype ST5, A8090) and VISA (genotype ST5, with agrC frame shift mutation, A8094)^27^ were sub-cultured onto brain heart infusion (BHI) agar (Oxoid). Single colonies were used to inoculate BHI broth (Oxoid) and grown at 37 °C with shaking overnight before use. *S. aureus* was grown in cation adjusted Muller Hinton Broth II (CAMHB, Becton Dickson) for microbroth dilution assays. All bacterial strains were stored in glycerol broth at −80 °C.

### Susceptibility testing

Antimicrobial activity of the individual peptides 3 and 5, and the mixtures were determined by a modified Clinical and Laboratory Standards Institute guidelines for microbroth dilution assays. Compounds stocks were prepared in water at each of the testing concentrations required and plated into 96 well flat bottom polypropylene microtiter plates (Greiner). Compounds were freeze dried in the plates. *S. aureus* (5 × 10^5^ CFU mL^−1^) was added to these plates in CAMHB (Becton Dickson), the plate covered with BreatheEasy membranes (Diversified Biotech) and absorbance of each well was measured at 595 nm every 4 h for 24 h (Clariostar plate reader, BMG). The percentage growth was calculated relative to the no compound control and the average of 3 biological replicates plotted using GraphPad Prism 8 software. The minimum inhibitory concentration (MIC) was determined by the lowest drug concentration that inhibited bacterial growth to 90% compared to the 0 mM control. While the IC_50_ was the concentration that inhibited 50% growth.

### Biofilm susceptibility assay

Sterile glass coverslips were coated with peptide mixtures of 3 and 5 by applying 100 μL of a 0.4 mM solution and allowing to dry overnight. An overnight culture of biofilm forming *S. aureus* (ATCC 29213) was used to inoculate tryptic soy broth with 1% glucose (TSB, Becton Dickinson) to approximately 10^7^ CFU mL^−1^. Into a 24-well non-treated plate containing a peptide coated coverslip per well, 1 mL of culture was added. The plate was incubated for 90 min with 100 rpm shaking at 37 °C, prior to washing the coverslips 3× in 2 mL PBS, by passing the slip through the liquid. Coverslips were placed into a new well with 1 mL fresh TSB, and incubated for 24 h with 100 rpm shaking at 37 °C. The coverslips were washed 3× in 2 mL PBS before placing them into 10 mL tube with 2 mL PBS. Bacteria were removed from the coverslip by 3 series of vortexing for 1 min, and bath sonicating for 1 min.^49^ Bacteria in this solution was determined by serial dilution and plating onto BHI for CFU enumeration. Data was plotted using GraphPad Prism 8 software.

### Cytotoxicity assay

Sterile glass coverslips were coated with peptide mixtures as described above and placed within a 96-well plate. HaCaT cells were seeded (50 000 cells per coverslip) in DMEM with 10% FBS. Cells were incubated for 48 hours at 37 °C, 5% CO_2._ The plate was aspirated and 80 μL of media was added to each well. 20 μL of MTS reagent (Promega, Madison, WI, USA) was then added and the plate was incubated at 37 °C, 5% CO_2_ for 2 hours. The plate was then read at 490 nm with a Clariostar plate reader (BMG labtech, Ortenberg, Germany).

### Confocal imaging of nanofibres with *S. aureus*

*S. aureus* strain (MRSA, A8090) was grown to mid-exponential phase, cells were stained for 15 min with Hoechst 33342 (ThermoFisher, 32 mM), washed and resuspended to 2 × 10^7^ CFU mL^−1^ in PBS as a 2× stock. Nanofibres were visualised by preparing peptides mixtures of 3 and 5 with 0.1% fluorescently labelled β^3^-C14 peptide (4). These were redissolved in water at a 2× concentrated (2 mM). 25 μL of both the peptide mixture and either *S. aureus* or PBS were added to a well of 8 well glass bottom culture slide (Corning). Fibres and *S. aureus* were imaged using Leica SP8 HyD system equipped with a 405 nm and 638 nm lasers and using a HC PLAPO CS2 40x/1.1NA water immersion lens (Leica, Mannheim, Germany). The 3D rendering software Imaris (v9.6.0. Bitplane, Zurich, Switzerland) was used to visualise the interaction the fibres had with *S. aureus* by applying a surface render to highlight nucleation around *S. aureus* – transparent surface options were used to visualise fibres wrapping around *S. aureus*.

### Transmission electron microscopy imaging of nanofibres with *S. aureus*

Transmission electron microscopy (TEM) imaging was used to analyse the morphology of the self-assembled peptide nanostructures. TEM was performed on a FEI Tecnai-12 EM equipped with a 4K CCD detector. Solutions of peptides and their mixtures were freshly prepared in water at 100 mM, incubated with MRSA and washed 3× in phosphate buffered saline pH 7, and a final wash in water. The solutions were loaded onto carbon film coated copper grids (400 mesh). The excess of sample was removed by blotting onto a filter paper and grids were allowed to dry at room temperature before collecting the images.

## Conflicts of interest

There are no conflicts to declare.

## Supplementary Material

NA-003-D0NA01018A-s001
