# Supplementary material for: Staphylococcus aureus entanglement in self-assembling β-peptide nanofibres decorated with vancomycin
Source: Nanoscale Adv. 2021 Mar 24;3(9):2607–16. doi: 10.1039/d0na01018a (PMC9419598; doi:10.1039/d0na01018a)
Supplement: NA-003-D0NA01018A-s001 [file NA-003-D0NA01018A-s001.pdf]

Electronic Supplementary Material (ESI) for Nanoscale Advances.
This journal is © The Royal Society of Chemistry 2021

Electronic Supplementary Information (ESI)

Staphylococcus aureus entanglement in self-assembling β-peptide nanofibres decorated with vancomycin

Jennifer A. E. Payne[1,2,3], Ketav Kulkarn[4], Thierry Izore[1,2], Alex J. Fulcher[5], Anton Y. Peleg[6], Marie-Isabel Aguilar[4], Max J. Cryle[1,2,3], Mark P. Del Borgo[7]

[1] Infection and Immunity Program, The Monash Biomedicine Discovery Institute, Department of Biochemistry and Molecular Biology, Monash University, Clayton, Victoria 3800, Australia.

[2] EMBL Australia, Monash University, Clayton, Victoria 3800, Australia

[3]The ARC Centre of Excellence for Innovations in Peptide and Protein Science, Monash University, Clayton, Victoria 3800, Australia

[4] Department of Biochemistry and Molecular Biology, Monash University, Clayton, Victoria 3800, Australia.

[5] Monash Micro Imaging, Monash University, Clayton, Victoria, 3800, Australia

[6] Infection and Immunity Program, Monash Biomedicine Discovery Institute and Department of Microbiology, Monash University, Clayton, Victoria, 3800, Australia and Department of Infectious Diseases, The Alfred Hospital and Central Clinical School, Monash University, Melbourne, Victoria, 3004, Australia.

[7] Department of Pharmacology, Monash University, Clayton, Victoria 3800, Australia.

Table of Contents

Synthesis 2

Synthesis of vancomycin β-peptide conjugate 2

Synthesis of functional lipidated tri-β-peptides 4

Synthesis of Lys-D-Ala-D-Ala variant 6

Peptide characterisation data 7

^{1}H NMR Spectra 7

Mass Spectra 11

HPLC traces 14

Video Files 15

Synthesis

Synthesis of vancomycin β-peptide conjugate

Propargyl amide vancomycin (1)

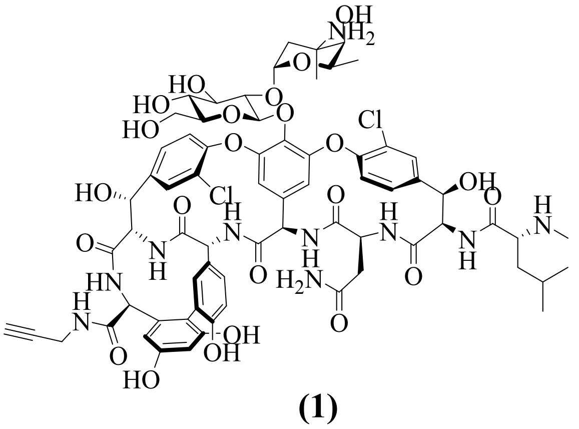

(1)

To vancomycin hydrochloride (1 g, 0.67 mmol) in anhydrous DMF (10 mL) was added DIPEA (~470 µL, 2.7 mmol), COMU (600 mg, 1.4 mmol) and propargyl amine (135 µL, 2.1 mmol). The reaction was stirred overnight at room temperature. The reaction mixture was then concentrated under vacuum and the residue was dissolved in 30% aq. CH_3CN (60 mL). The crude product was purified by injecting the sample (~7.5 mL) onto a reverse-phase preparative column, eluted over a 55 min gradient from 0 to 50 % solvent B, (solvent A: 0.1 % TFA/H2O; solvent B: 0.1 % TFA/CH_3CN) with a flow rate of 6 mL/min. The fractions were collected and analysed for purity by injecting the samples onto a reverse-phase analytical column, eluted over a 45 min gradient from 0 to 45 % solvent B, with a flow rate of 1 mL/min. The pure fractions were pooled to provide the propargyl amide vancomycin trifluoroacetate **1** (180 mg, 19 % yield) after the recovery of starting material (100 mg).

Synthesis of tri β-peptide AcβSerAzAlaOH (2)

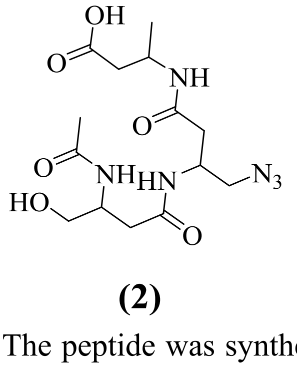

(2)

The peptide was synthesised on a 0.25 mmol scale using standard Fmoc chemistry on Wang resin. The resin was swollen in DMF (2 mL) and then soaked in Fmoc-protected β amino acid (2.1 eq. to resin loading), dissolved in DMF (2 mL) along with HBTU (2 eq. to resin loading),

HOBt (2 eq. to resin loading), DMAP (10 mol %) and DIPEA (4 eq. to resin loading), overnight with gentle agitation. The resin was thoroughly washed with DMF (3 x 4 mL) and the Fmoc protecting group on the amino acid was removed by soaking the resin twice in 20 % piperidine, with 0.1 M HOBt, in DMF (4 mL) for 15 minutes each. The resin was washed with DMF (3 x 4 mL), soaked in Fmoc-protected amino acid (2.1 eq. to resin loading), dissolved in DMF (4 mL) along with HBTU (2 eq. to resin loading), HOBt (2 eq. to resin loading) and DIPEA (4 eq. to resin loading), for 2 hours. β Peptide elongation cycle was then repeated until the sequence was complete. After removing the terminal Fmoc protecting group on the peptide, the resin was treated with a solution of 10 % v/v acetic anhydride and 2.5 % v/v DIPEA in DMF (4 mL) for 30 minutes to afford an acetyl-capped N-terminus. The resin was washed with DMF (2 x 4 mL), CH_2Cl_2 (2 x 4 mL), Et_2O (2 x 4 mL) and air dried. Cleavage was performed on the resin (0.25 mmol), by treating the resin with a cleavage solution (10 mL) comprising of H_2O (2.5 % v/v), triisopropylsilane (2.5 % v/v) in CF_3COOH, for 2 hours. CF_3COOH was then evaporated under a stream of N_2 and the peptide was precipitated by addition of Et_2O (50 mL). The precipitate was filtered and dissolved in 50 % aqueous CH_3CN for lyophilisation. The peptide was redissolved in H_2O (5 mL) and purified by injecting the sample onto a reverse-phase preparative column, eluted over a 55 min gradient from 0 to 40 % solvent B, (solvent A: 0.1 % TFA/H_2O; solvent B: 0.1 % TFA/CH_3CN) with a flow rate of 6 mL/min. The fractions were analysed for purity by injecting the samples onto a reverse-phase analytical column, eluted over a 45 min gradient from 0 to 25 % solvent B, with a flow rate of 1 mL/min. Pure fractions were pooled to afford peptide AcβSerAzAlaOH **2** (40 mg, 43 % yield).

Synthesis of clicked product AcβSerAz(Vancomycin)βAlaOH (3)

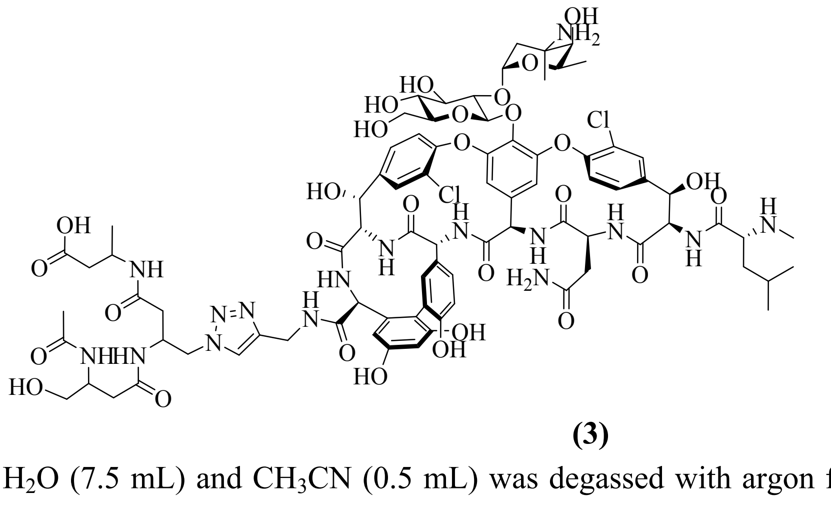

(3)

H_2O (7.5 mL) and CH_3CN (0.5 mL) was degassed with argon for 5 min. To this was added $NaHCO_3$ (32 mg) ascorbic acid (26 mg), propargyl vancomycin trifluoroacetate **1** (24 mg,

15 μmol) and AcβSerAzAlaOH **2** (7 mg, 19 μmol). $CuSO_4.5H_2O$ (16 mg, 64 μmol) was then added to the reaction solution and vortexed until completely dissolved. The reaction was gently agitated for 60 min. The crude product was purified by injecting it onto a reverse-phase preparative column, eluted over a 55 min gradient from 0 to 55 % solvent B, (solvent A: 0.1 % TFA/H_2O; solvent B: 0.1 % TFA/CH_3CN) with a flow rate of 6 mL/min. The fractions were collected and analysed for purity by reverse-phase analytical HPLC, eluted over a 45 min gradient from 0 to 45 % solvent B, with a flow rate of 1 mL/min. Pure fractions were pooled to provide AcβSerineTriazole(Vancomycin)βAlanine trifluoroacetate **3** (21 mg, 71 % yield).

Synthesis of functional lipidated tri-β-peptides

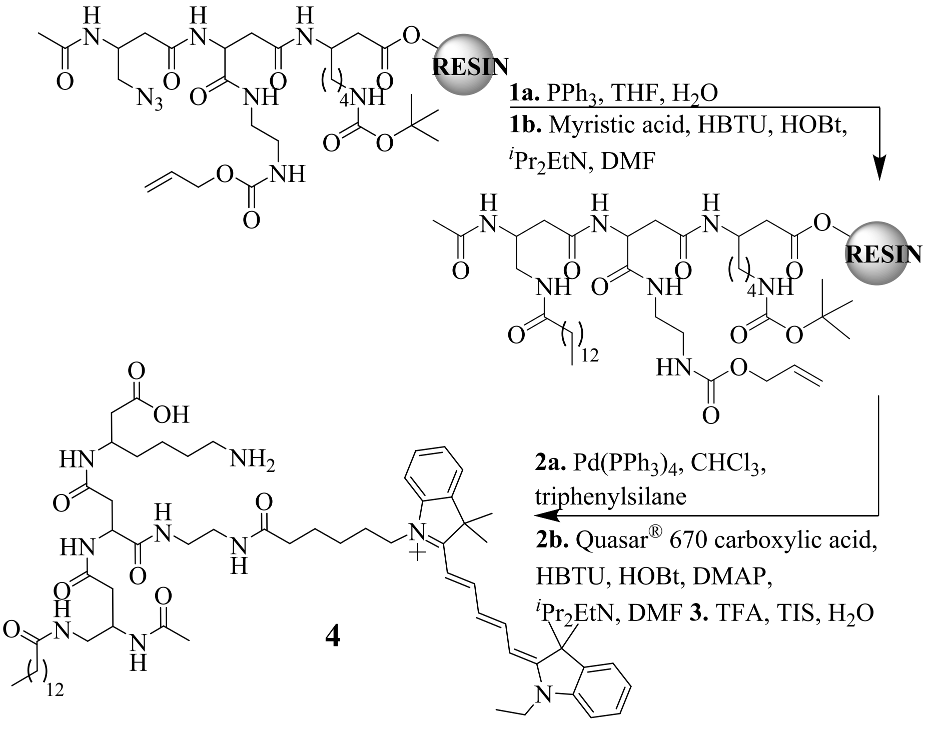

Scheme S1: Synthesis of lipidated tri β-peptide conjugated to fluorophore AcβAlaC14Cy5βLysOH **(4)**

The peptide was synthesised on a 0.1 mmol scale using standard Fmoc chemistry on chlorotrityl resin (0.3 mmol/g loading). The resin was swollen in DCM (2 mL) and then soaked in Fmoc-protected β amino acid (2 eq. to resin loading), dissolved in DCM (2 mL) along with DIPEA (3 eq. to resin loading), overnight with gentle agitation. The resin was thoroughly washed with DMF (3 x 3 mL) and the Fmoc protecting group on the amino acid was removed by soaking the resin twice in 20 % piperidine, with 0.1 M HOBt, in DMF (3 mL) for 15 minutes each. The resin was washed with DMF (3 x 3 mL), soaked in Fmoc-protected amino acid (3 eq. to resin loading), dissolved in DMF (3 mL) along with HBTU (3 eq. to resin loading), HOBt (3 eq. to resin loading) and DIPEA (4 eq. to resin loading), for 2 hours. β Peptide elongation cycle was

then repeated until the sequence was complete. After removing the terminal Fmoc protecting group on the peptide, the resin was treated with a solution of 10 % v/v acetic anhydride and 2.5 % v/v DIPEA in DMF (4 mL) for 30 minutes to afford an acetyl-capped N-terminus. The resin was washed with DMF (2 x 3 mL), CH_2Cl_2 (2 x 3 mL), Et_2O (2 x 4 mL), air dried for 10 minutes, and transferred to a 50 mL vial for further manipulation.

Derivatisation of the *N*-acetyl β^3-tripeptide, on solid support, was preceded by the reduction of the azido-alanine residue on the β^3-peptide. The resin (0.1 mmol) was swollen in THF (2.5 mL) and then soaked in a solution of PPh_3 (4 eq. to resin loading), in THF (2 mL) and H_2O (50 µL), overnight with gentle agitation. The resin was filtered through a sintered glass funnel and washed with THF (2 x 3 mL) and DMF (2 x 3 mL). The resin was soaked in myristic acid (3 eq. to resin loading), dissolved in DMF (4 mL) along with HBTU (3 eq. to resin loading), HOBt (3 eq. to resin loading) and DIPEA (4 eq. to resin loading), for 1 h. The resin was subsequently washed with DMF (2 x 3 mL), CH_2Cl_2 (2 x 3 mL), Et_2O (2 x 4 mL), air dried for 10 min and continued with further chemical manipulation to afford AcβAlaC14Cy5βLysOH **(4)** or cleaved to afford AcβAlaC14βLysβAlaOH **(5)**.

To a 50 mL vial was added $CHCl_3$ (10 mL) which was rigorously degassed by bubbling a stream of argon. A portion of the degassed $CHCl_3$ (~2 mL) was then used to swell the resin (0.1 mmol), contained in a separate 15 mL vial pre-purged of air with argon. $PhSiH_3$ (250 µL) was added to the remaining $CHCl_3$ (~4 mL) whilst still bubbling with a stream of argon. $Pd(PPh_3)_4$ (250 mg) was then added and the mixture was shaken until a homogeneous solution was achieved. The resin was then soaked in the $Pd(PPh_3)_4$ solution for 2 hours, with gentle agitation, filtered through a sintered glass funnel, and washed with CH_2Cl_2 (3 x 3 mL) and DMF (3 x 3 mL) to remove the catalyst. The resin was then soaked in Quasar® 670 carboxylic acid (1 eq. to resin loading), dissolved in DMF (2 mL) along with HBTU (1 eq. to resin loading), HOBt (1 eq. to resin loading), DMAP (0.1 eq. to resin loading) and DIPEA (2.5 eq. to resin loading), for 2 hours with occasional stirring and excluded from light. The resin was subsequently washed with DMF (2 x 3 mL), CH_2Cl_2 (2 x 3 mL), Et_2O (2 x 4 mL), air dried and subsequently cleaved from the resin.

Cleavage was performed on the resin (0.1 mmol), by treating the resin with a cleavage solution (10 mL) comprising of H_2O (2.5 % v/v), triisopropylsilane (2.5 % v/v) in CF_3COOH, for 2 hours. CF_3COOH was then evaporated under a stream of N_2 and the peptide was precipitated

by addition of Et_2O (50 mL). The precipitate was filtered and dissolved in 50 % aqueous CH_3CN for lyophilisation. The peptide was redissolved in 60-70 % aqueous CH_3CN (5 mL) and purified by injecting the sample onto a reverse-phase preparative column, eluted over a 55 min gradient from 20 to 85 % solvent B, (solvent A: 0.1 % TFA/H_2O; solvent B: 0.1 % TFA/CH_3CN) with a flow rate of 6 mL/min. The fractions were collected and analysed for purity by injecting the samples onto a reverse-phase analytical column, eluted over a 45 min gradient from 0 to 70 % solvent B, (solvent A: 0.1 % TFA/H_2O; solvent B: 0.1 % TFA/CH_3CN) with a flow rate of 1 mL/min. The pure fractions were pooled to afford AcβAlaC14Cy5βLysOH trifluoroacetate **4** (13 mg, 10 % yield) or AcβAlaC14βLysβAlaOH trifluoroacetate **5** (30 mg, 42 % yield).[1,2]

Synthesis of Lys-D-Ala-D-Ala variant

Synthesis of Gly-Gly-Gly-Lys-D-Ala-D-Ala (6)

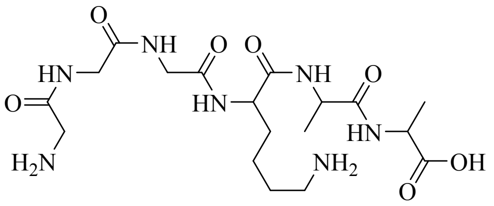

(6)

The peptide was synthesised on a 0.10 mmol scale using standard Fmoc chemistry on Wang resin as described above. The peptides were then cleaved and purified by injecting the them onto a reverse-phase preparative column, eluted over a 55 min gradient from 0 to 20 % solvent B (solvent A: 0.1 % TFA/H_2O; solvent B: 0.1 % TFA/CH_3CN) with a flow rate of 6 mL/min. The pure fractions were pooled to afford Gly-Gly-Gly-Lys-D-Ala-D-Ala trifluoroacetate **(6)** (23 mg, 33 % yield).

Peptide characterisation data

^{1}H NMR Spectra

^{1}H NMR of propargyl Vancomycin (1)

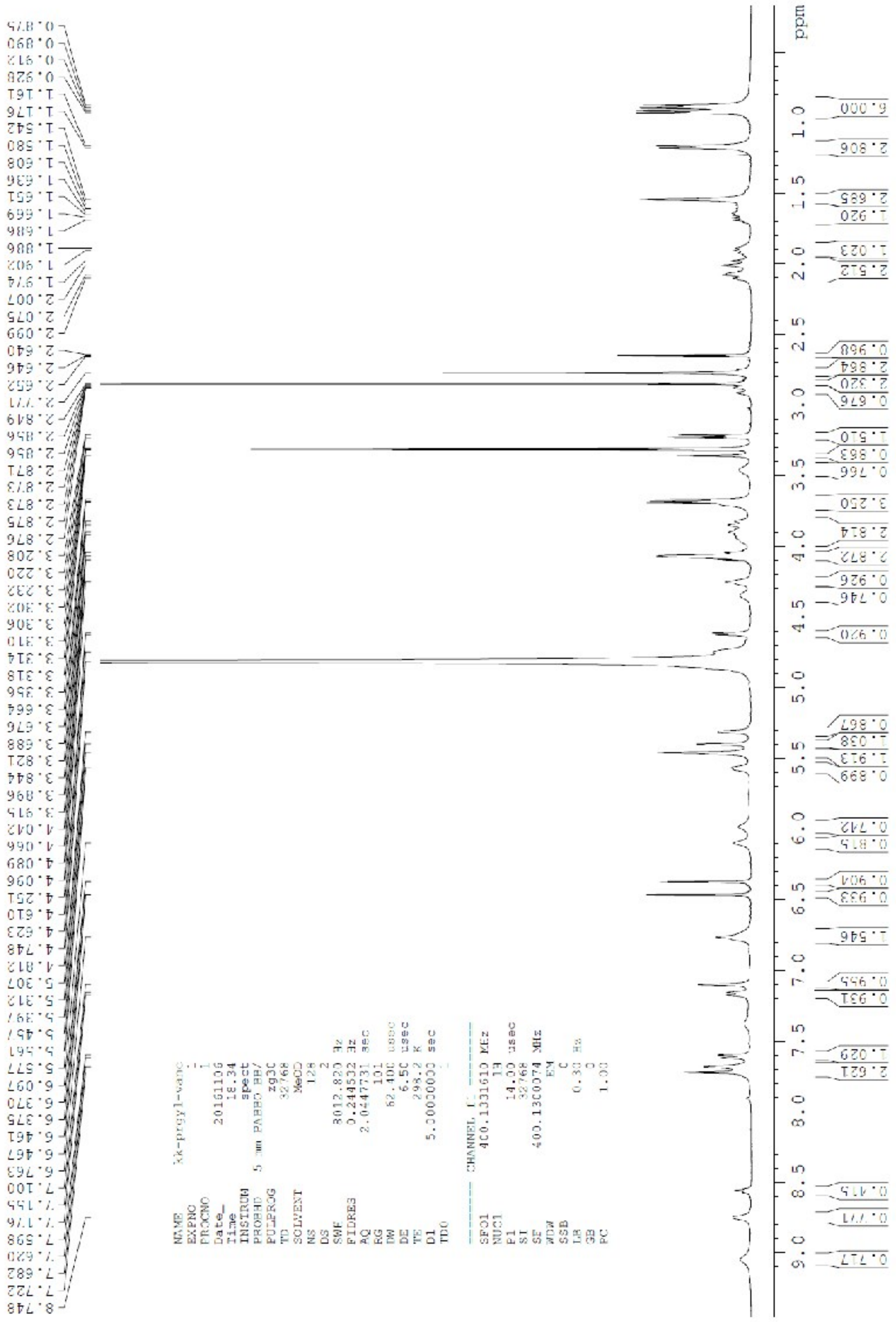

^{1}H NMR of β tri-peptide AcβSerAzAlaOH (2)

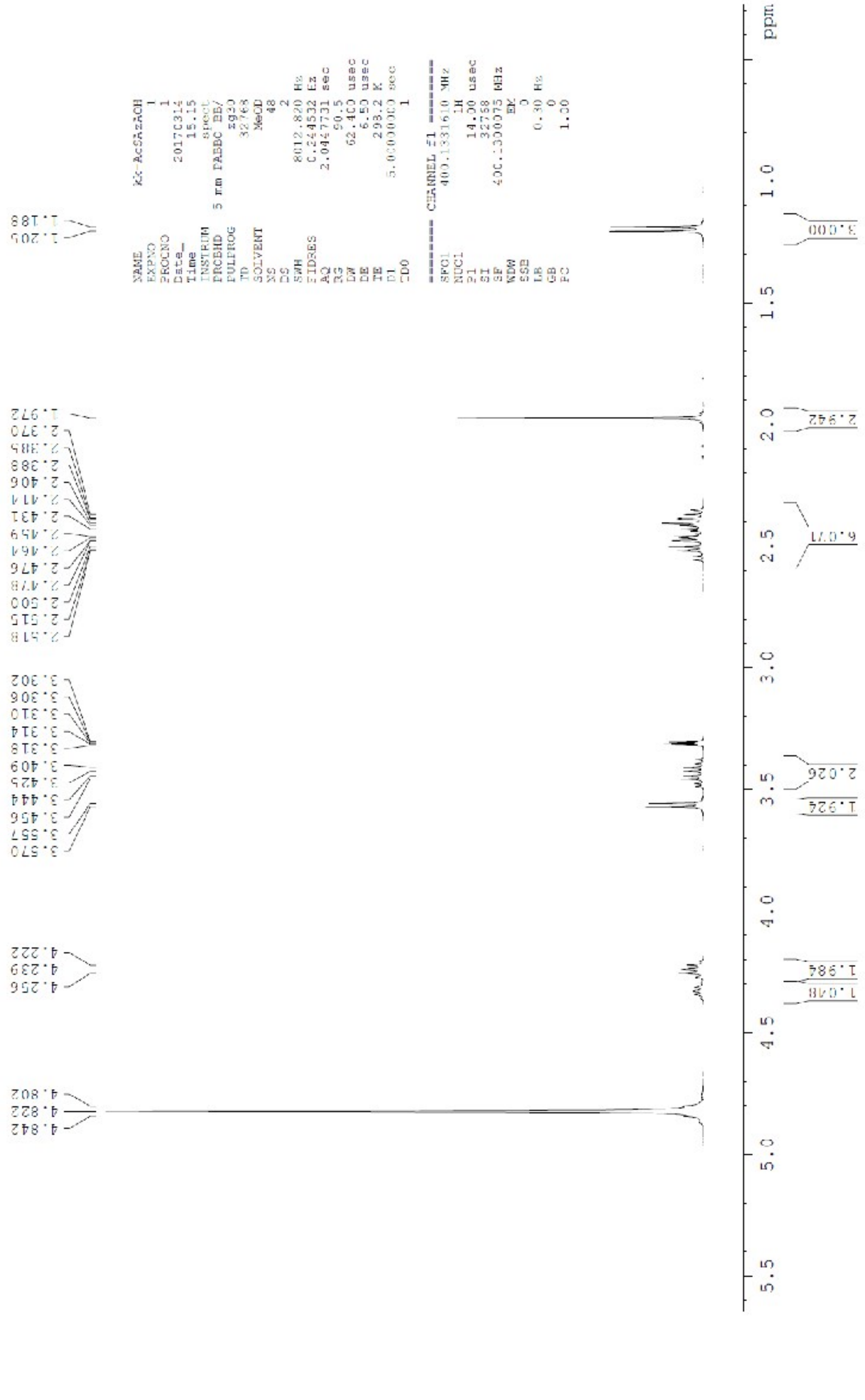

^{1}H NMR of clicked product AcβSerAz(Vancomycin)βAlaOH (3)

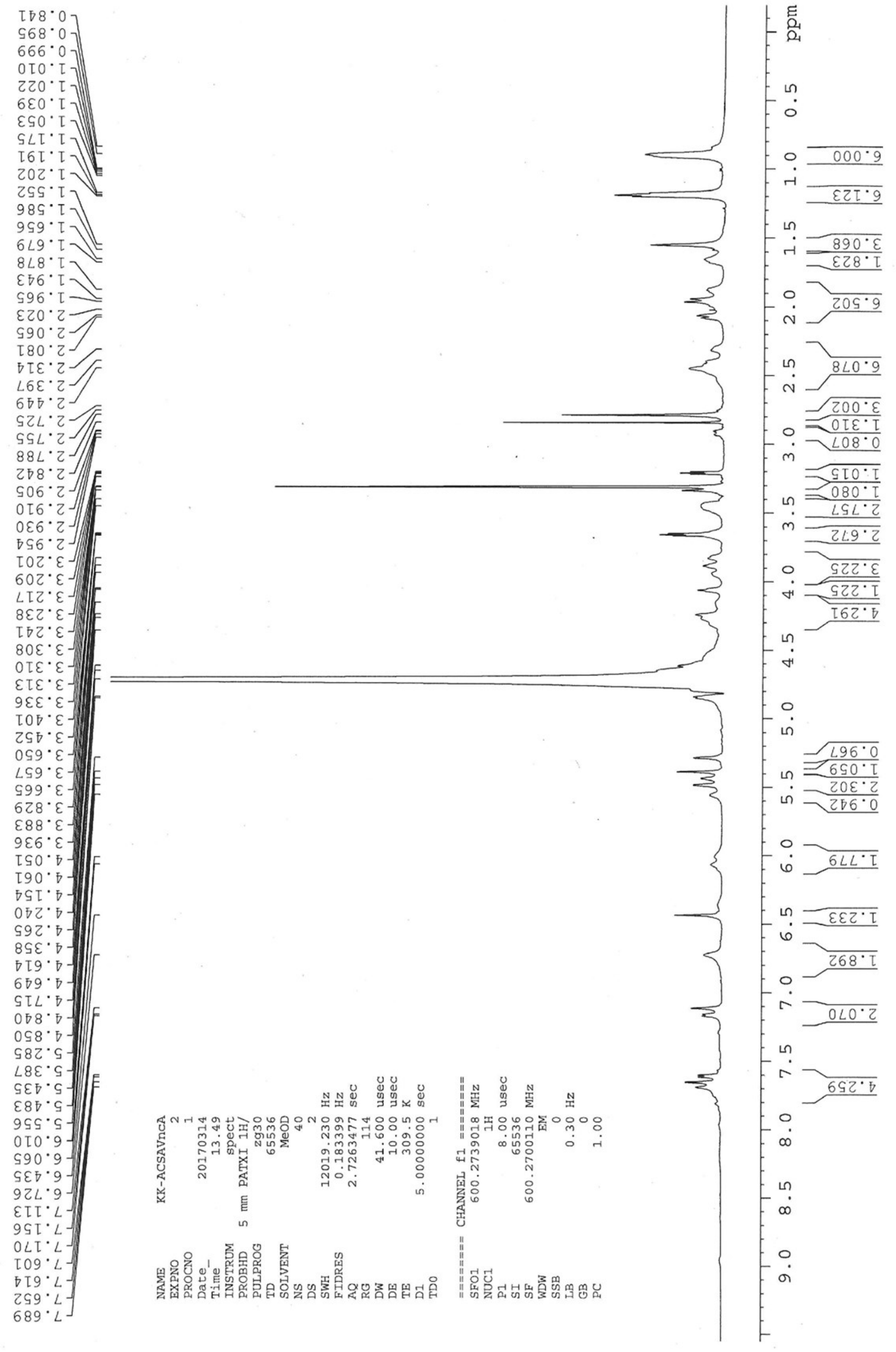

¹H NMR stack of AcβSerAzAlaOH (1), propargyl Vancomycin (2) and AcβSerAz(Vancomycin)βAlaOH (3)

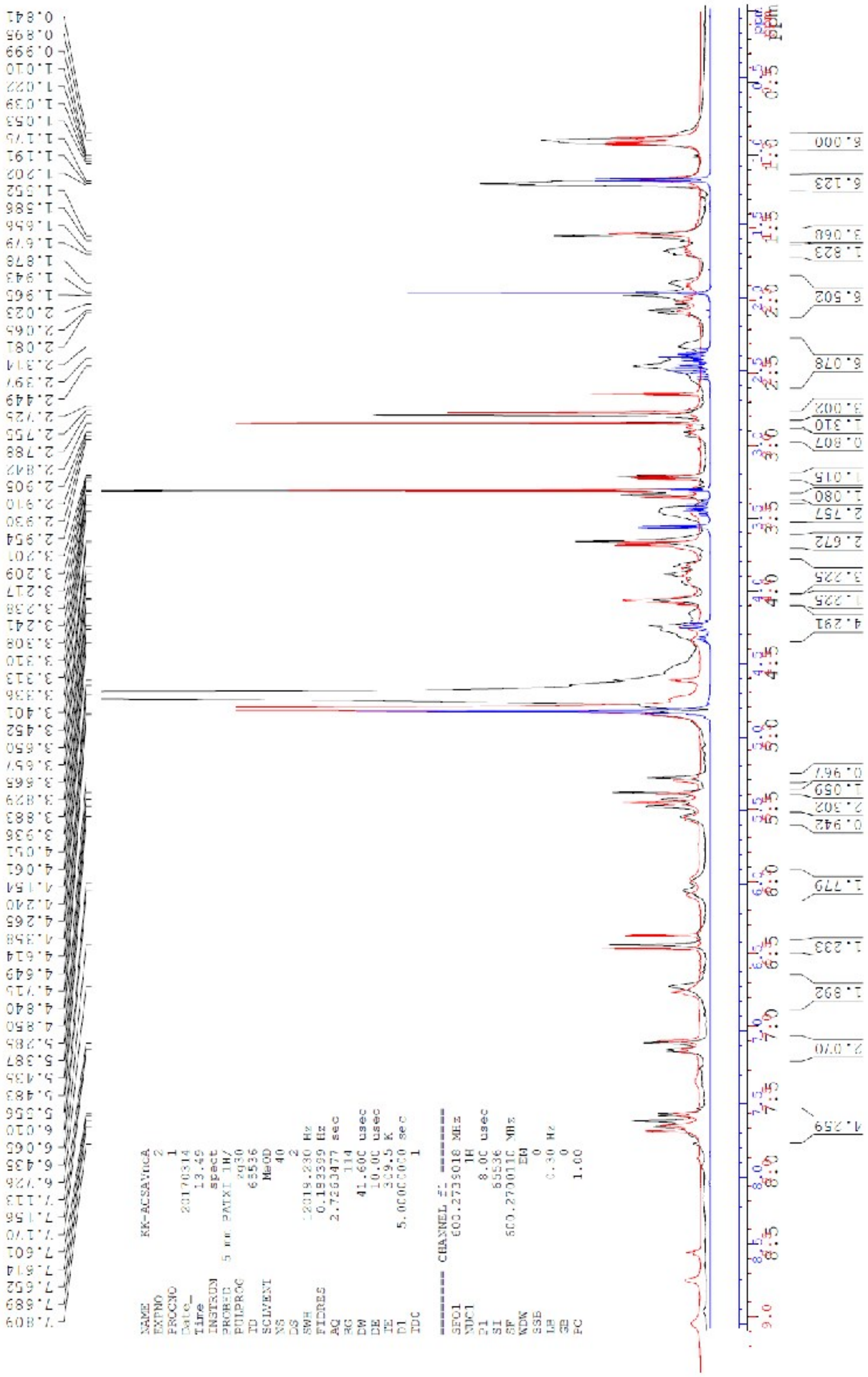

Mass Spectra

Accurate MS analysis of propargyl Vancomycin (1)

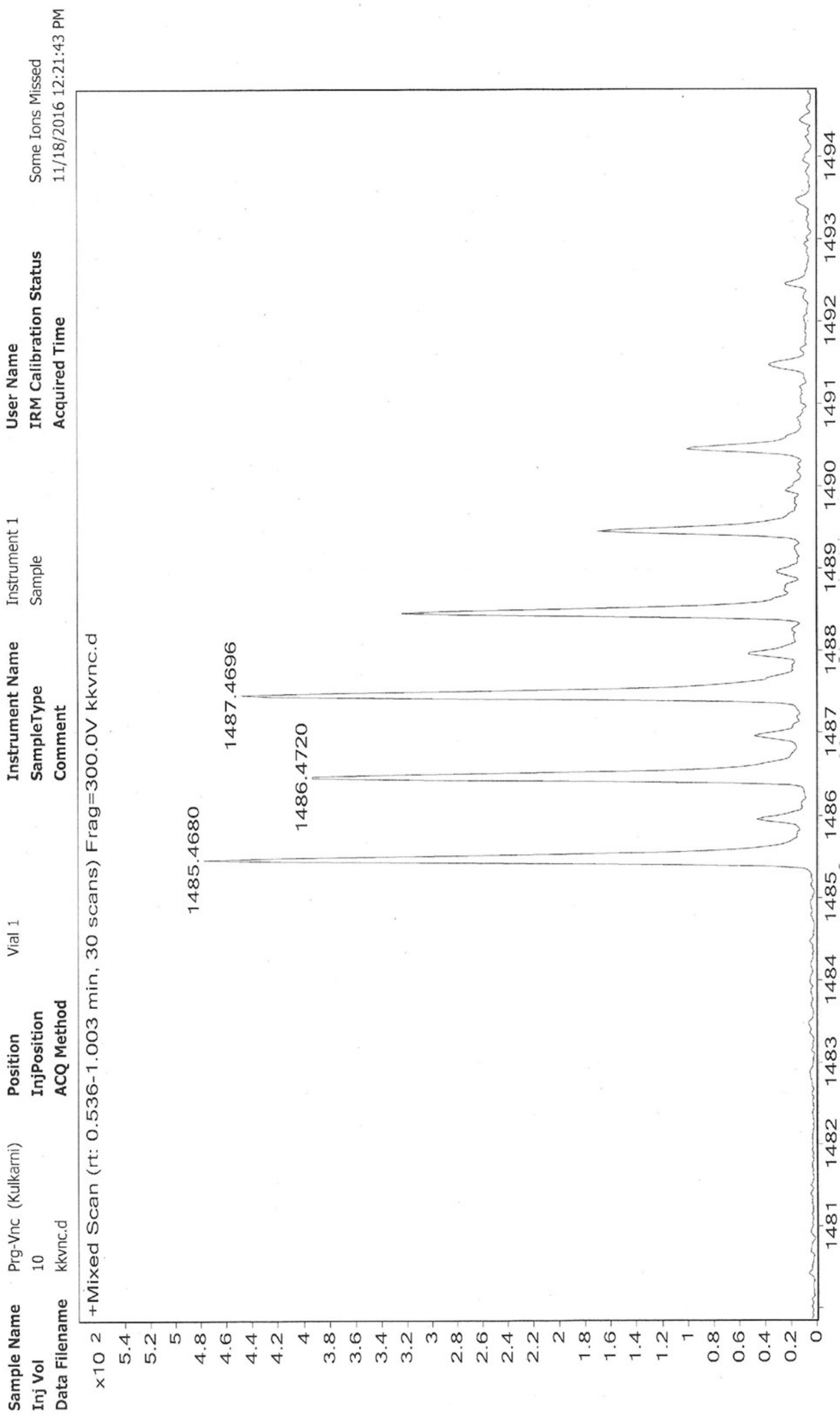

MS analysis of AcβSerAzAlaOH (2)

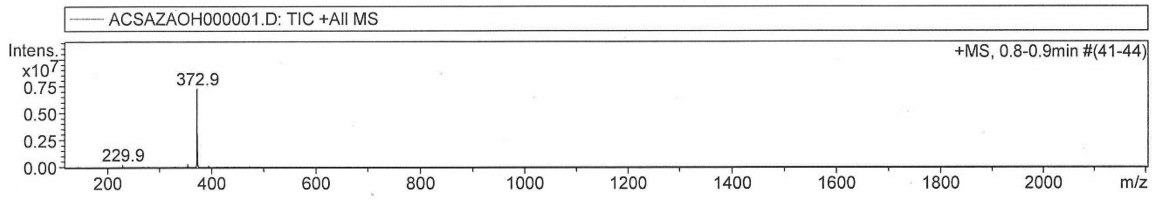

Accurate MS analysis of clicked product AcβSerAz(Vancomycin)βAlaOH (3)

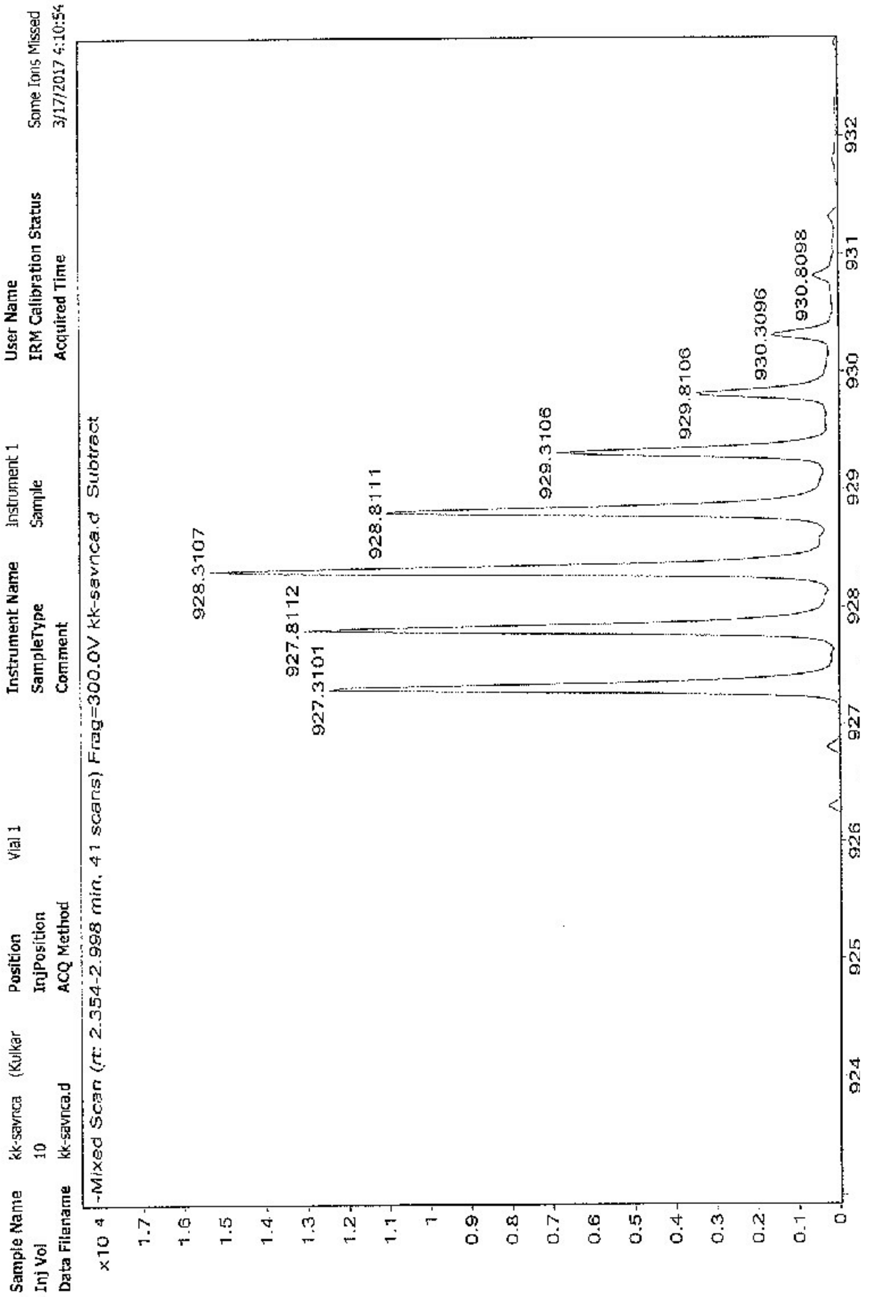

Accurate MS analysis of clicked product AcβSerAz(Vancomycin)βAlaOH (3)

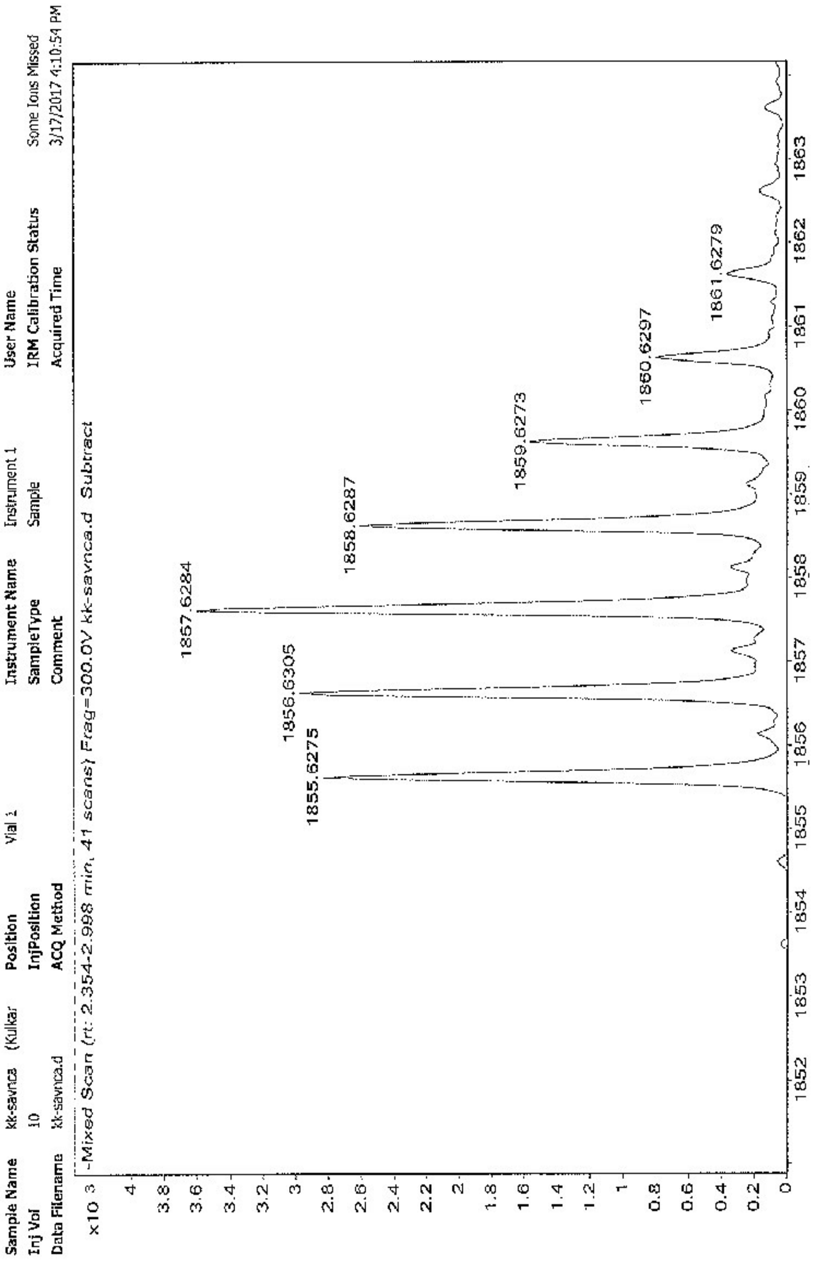

MS analysis of AcβAlaC14Cy5βLysOH (4)

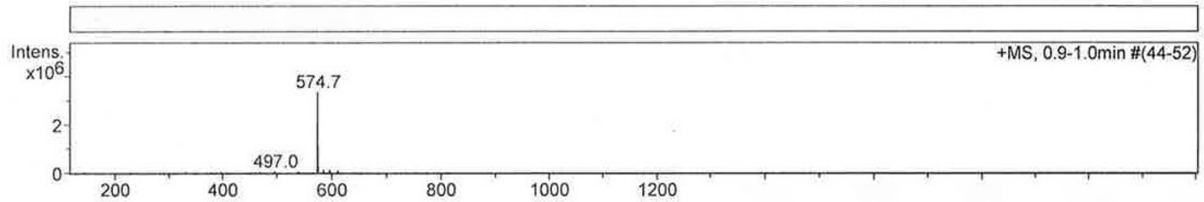

MS analysis of AcβAlaC14βLysβAlaOH (5)

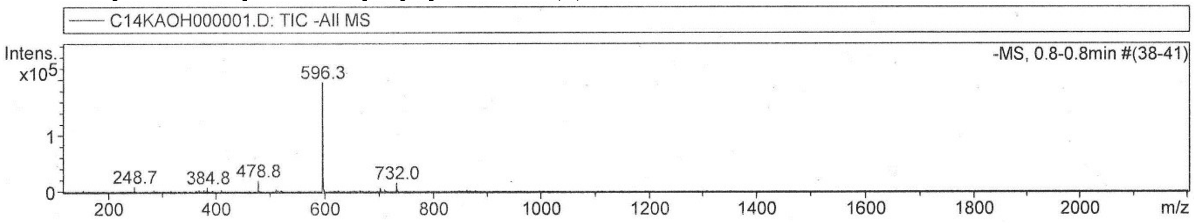

MS analysis of GlyGlyGlyLysdAladAla (6)

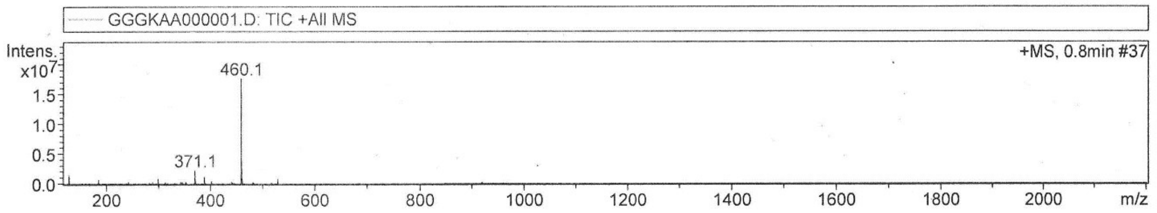

HPLC traces

HPLC analysis of propargyl Vancomycin (1)

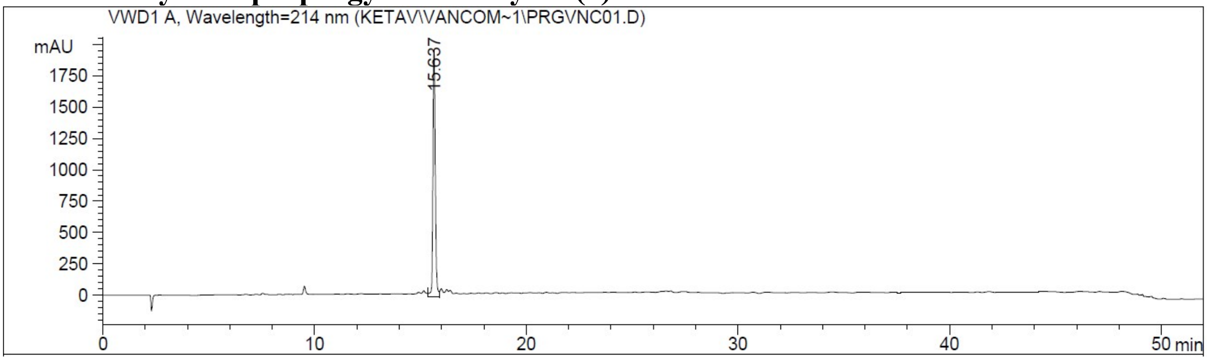

HPLC analysis of AcβSerAzAlaOH (2)

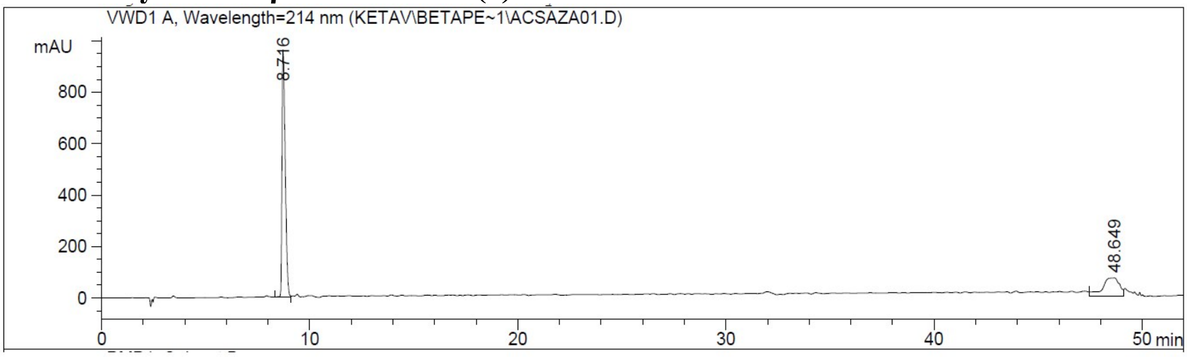

HPLC analysis of clicked product AcSerAz(Vancomycin)AlaOH (3)

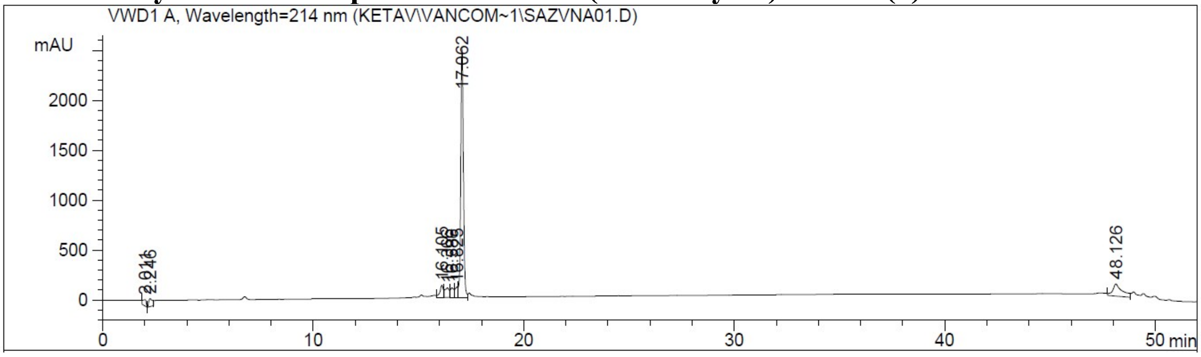

HPLC analysis of AcβAlaC14Cy5βLysOH (4)

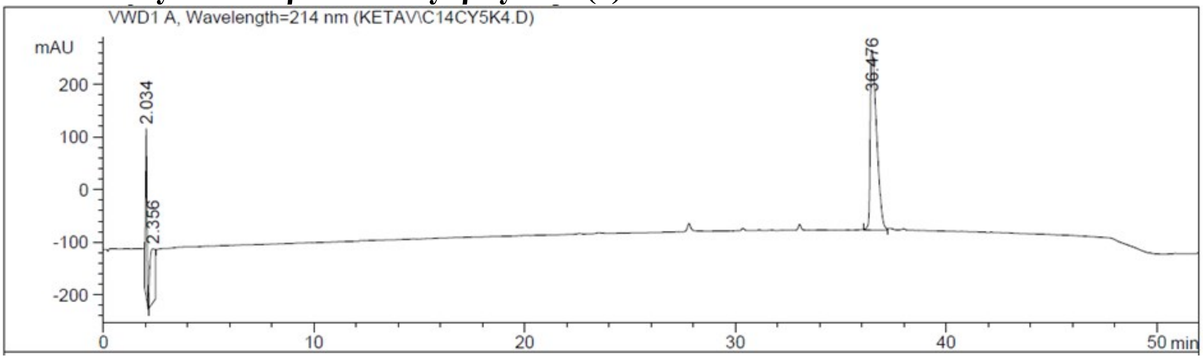

HPLC analysis of AcβAlaC14βLysβAlaOH (5)

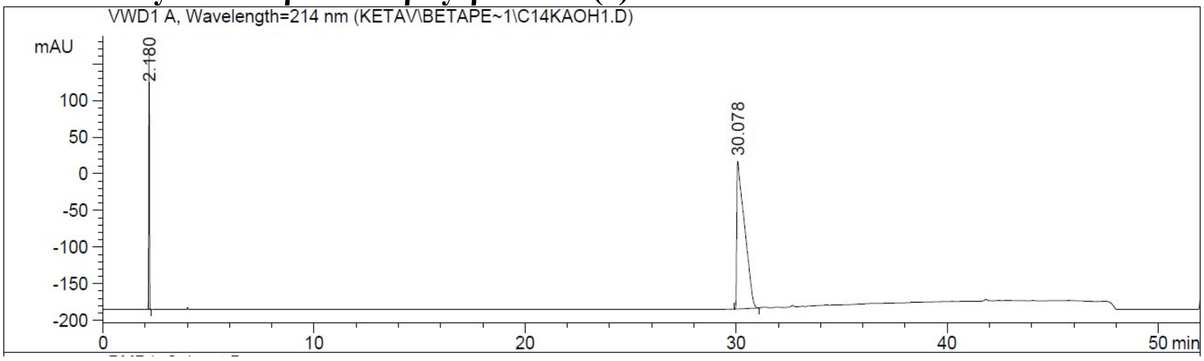

Video Files

Link for ESI Video 1 can be found HERE

Link for ESI Video 2 can be found HERE
